# The presence of *Giardia intestinalis* in donkeys, *Equus asinus*, in China

**DOI:** 10.1186/s13071-016-1936-0

**Published:** 2017-01-03

**Authors:** Xiao-Xuan Zhang, Fu-Kai Zhang, Fa-Cai Li, Jun-Ling Hou, Wen-Bin Zheng, Shuai-Zhi Du, Quan Zhao, Xing-Quan Zhu

**Affiliations:** 1 State Key Laboratory of Veterinary Etiological Biology, Key Laboratory of Veterinary Parasitology of Gansu Province, Lanzhou Veterinary Research Institute, Chinese Academy of Agricultural Sciences, Lanzhou, Gansu Province 730046 People’s Republic of China; 2 College of Animal Science and Technology, Jilin Agricultural University, Changchun, Jilin Province 130118 People’s Republic of China; 3 Qilu Animal Health Products Co., Ltd., Jinan, Shandong Province 250100 People’s Republic of China

**Keywords:** *Giardia intestinalis*, Prevalence, Genotyping, Donkey, China

## Abstract

**Background:**

*Giardia intestinalis* is one of the most important zoonotic enteric parasites. As no information regarding prevalence and genotype of *G. intestinalis* in donkeys (*Equus asinus*) in China is available, 181 faecal samples from 48 donkeys from Jilin Province, from 104 from Shandong Province and from 29 from Liaoning Province were examined between May and December 2015.

**Findings:**

Twenty-eight (15.47%) out of 181 donkey samples were tested *G. intestinalis*-positive by nested amplification of the triosephosphate isomerase (*tpi*) gene. The prevalence in different regional groups varied from 10.42 to 18.27%. The prevalence in adult and young donkeys was 14.29 and 22.92%, respectively. Otherwise, the prevalence was 11.69% in summer and 18.27% in winter. However, no statistically significant differences were found in relation to region or age group. Sequence analysis of the *tpi*, glutamate dehydrogenase (*gdh*) and beta giardin (*bg*) loci identified 4, 1 and 3 subtypes of assemblage B, respectively. Moreover, four novel multilocus genotypes (MLGs novel-1 to novel-4) were identified in assemblage B.

**Conclusions:**

This first report of *G. intestinalis* in donkeys in China indicates that further studies of nation-wide molecular epidemiology and geographical distribution of *Giardia* in donkeys are warranted. Effective strategies should be implemented to control *G. intestinalis* infection in donkeys, other animals and humans.

**Electronic supplementary material:**

The online version of this article (doi:10.1186/s13071-016-1936-0) contains supplementary material, which is available to authorized users.

## Background

*Giardia intestinalis* is the only species of *Giardia* which is found in human beings [1–4]. It is not only distributed worldwide, but also can infect many vertebrates [5, 6]. Eight assemblages (A-H) have been identified within *G. intestinalis* [6–8]. Of these, assemblages C-H seem to be animal-specific [9], but assemblages A and B can infect humans and a wide range of non-human hosts [6, 10]. Diarrhea is the main symptom of giardiasis [11] and transmission is mainly through ingestion of *Giardia* cysts in contaminated food or water [12]; approximately 2.8 × 10^8^ cases of human giardiasis are reported world-wide per year, and the majority of them are reported in developing countries [6]. In view of such a serious situation, giardiasis has attracted considerable attention around the world. Although *G. intestinalis* infections have been reported in humans and a variety of animal species [13–16], there is little information in donkeys (*Equus asinus*).

*Giardia intestinalis* infection in horses has been reported in many countries around the world including China [17–21]. The donkey belongs to the genus *Equus*. It is an important edible animal species and used in Chinese traditional medicine, and is closely related to horses. Because they are maintained in a close association with their owners and veterinary personnel, donkeys are the important reservoirs for transmission of pathogens (such as *Cryptosporidium hominis* and *Toxoplasma gondii*) to humans and other animals [22, 23]. To determine whether donkeys are hosts of *G. intestinalis*, we conducted a study on the prevalence and genotypes of *G. intestinalis* in donkeys in Jilin, Liaoning and Shandong Provinces, China.

## Methods

### Collection and preparation of faecal samples

A total of 181 donkey faecal samples (48 from Jilin, 27 from Liaoning and 104 from Shandong) were collected from three provinces, in northeastern and eastern China, between March and December 2015. Each of the fresh faecal samples was collected into sterile gloves separately after its defecation onto the ground, placed into box with ice and transported to the laboratory immediately. Genomic DNA was extracted directly from each faecal sample using the Stool DNA kit (OMEGA, Norcross, Georgia, USA) according to the manufacturer’s instructions. Genomic DNA was stored at -20 °C until PCR amplification.

### PCR amplification

The prevalence and genotypes of *G. intestinalis* were determined by the nested PCR amplification of the triosephosphate isomerase (*tpi*) gene, beta giardin (*bg*) gene and glutamate dehydrogenase (*gdh*) genes as described by Zhao et al. [24]. The primers and their annealing temperatures are listed in Table 1. Positive and negative controls were included in each amplification. Amplification products were observed under UV light after electrophoresis in 1.5% agarose gels containing GoldView™ (Solarbio, Beijing, China).

**Table 1:** Primers used in the study, annealing temperatures used in the PCRs and expected sizes of the PCR products

Gene	Primer	Sequence (5'-3')	Annealing temperature (°C)	Fragment length (bp)	Reference
*tpi*	F1	AAATIATGCCTGCTCGTCG	55	530	[24]
	R1	CAAACCTTITCCGCAAACC			
	F2	CCCTTCATCGGIGGTAACTT	55		
	R2	GTGGCCACCACICCCGTGCC			
*gdh*	F1	TTCCGTRTYCAGTACAACTC	50	530	[24]
	R1	ACCTCGTTCTGRGTGGCGCA			
	F2	ATGACYGAGCTYCAGAGGCACGT	65		
	R2	GTGGCGCARGGCATGATGCA			
*bg*	F1	AAGCCCGACGACCTCACCCGCAGTGC	50	511	[24]
	R1	GAGGCCGCCCTGGATCTTCGAGACGAC			
	F2	GAACGAACGAGATCGAGGTCCG	60		
	R2	CTCGACGAGCTTCGTGTT			

### Sequence and phylogenetic analyses

Positive secondary PCR products were sequenced by Genscript Company (Nanjing, China). Bidirectional sequencing was used to confirm the accuracy of the sequences. Sequences with mutations were considered as novel genotypes when confirmed from independent two PCR reactions on the same sample. To identify the assemblages and subtypes, nucleotide sequences were aligned with known reference *tpi*, *gdh* and *bg* gene sequences of *G. intestinalis* available in GenBank using the BLAST (http://www.ncbi.nlm.nih.gov/BLAST/) and computer program Clustal X 1.83.

### Statistical analysis

The relationship between prevalence of *G. intestinalis*-infected donkeys and different variables including age, geographic origin and seasons were analyzed by Chi-square test using SPSS version 17.0 (SPSS Inc., Chicago, IL, USA) [25]. The results were considered significant statistically if *P* < 0.05. Odds ratios (ORs) and their 95% confidence intervals (CI) are also given.

## Results and discussion

In this study, a total of 28 (15.47%, 95% CI: 10.20–20.74) out of 181 donkey samples were PCR-positive for *G. intestinalis* (Table 2). The prevalence was 22.92% (95% CI: 11.03–34.81) in young donkeys and 14.29% (95% CI: 8.34–20.23) in adults; no significant difference was observed (*χ* ^2^ = 1.90, *df* = 1, *P* = 0.17) (Table 2). The prevalence in summer and winter was 11.69% (95% CI: 4.51–18.87) and 18.27% (95% CI: 10.84–25.70), respectively (*χ* ^2^ = 1.47, *df* = 1, *P* = 0.23) (Table 2). Donkeys from Jilin Province (5/48, 10.42%, 95% CI: 1.78–19.06) had a lower prevalence than those from Shandong Province (19/104, 18.27%, 95% CI: 10.84–25.70) and Liaoning Province (4/29, 13.79%, 95% CI: 1.24–26.34); however, these differences were not significant (*χ* ^2^ = 1.62, *df* = 2, *P* = 0.44) (Table 2). Moreover, *G. intestinalis* prevalence in different farms ranged from 6.12 to 29.09% (Table 3). Sequences analysis of the *tpi*, *gdh* and *bg* loci indicated only assemblage B was found in the present study (Additional file 1: Figure S1).

**Table 2:** Factors associated with prevalence of *Giardia intestinalis* in donkeys in northern China

Factor	Category	No. tested	No. positive	% (95% CI)	*P*-value	OR (95% CI)
Region	Jilin Province	48	5	10.42 (1.78–19.06)	0.44	Reference
	Liaoning Province	29	4	13.79 (1.24–26.34)		1.38 (0.34–5.60)
	Shandong Province	104	19	18.27 (10.84–25.70)		1.92 (0.67–5.50)
Age	Adult	133	19	14.29 (8.34–20.23)	0.17	Reference
	Young	48	11	22.92 (11.03–34.81)		1.33 (0.58–3.07)
Season	Summer	77	9	11.69 (4.51–18.87)	0.23	Reference
	Winter	104	19	18.27 (10.84–25.70)		1.69 (0.72–3.97)
Total		181	28	15.47 (10.20–20.74)

**Table 3:** *Giardia intestinalis* genotypes identified in donkeys in different farms

Region	Farm ID	Age category (*n*)	No. positive/No. tested (%)	Genotype (*n*)
Jilin Province	Farm 1	Young (10); Adult (38)	5/48 (10.42)	BIV-1 (*n* = 2); BIV-novel-2 (*n* = 2); BIV-novel-3 (*n* = 1)
Liaoning Province	Farm 2	Young (6); Adult (23)	4/29 (13.79)	BIV-1 (*n* = 2); BIV-novel-3 (*n* = 1); BIV-novel-4 (*n* = 1)
Shandong Province	Farm 3	Young (14); Adult (35)	3/49 (6.12)	BIV-1 (*n* = 1); BIV-novel-4 (*n* = 2)
	Farm 4	Young (18); Adult (37)	16/55 (29.09)	BIV-1 (*n* = 12); BIV-novel-3 (*n* = 4)
Total		Young (48); Adult (133)	28/181 (15.47)	BIV-1 (*n* = 17); BIV-novel-2 (*n* = 2); BIV-novel-3 (*n* = 6); BIV-novel-4 (*n* = 3)

The overall prevalence of *G. intestinalis* infection in donkeys was 15.47% (28/181, 95% CI: 10.20–20.74), which was higher than that in horses in Xinjiang (1.5%) [17], Brazil (0.5%) [26], foals in Belgium (14.2%) [20], Germany (10%) [20], Greece (11.6%) [20], the Netherlands (11.4%) [20] and Italy (8.6%) [18], but lower than that in horses in Colombia (17.4%) [19]. Previous studies demonstrated that survival of *G. intestinalis* is more likely to be affected by climate (temperature and relative humidity) [6, 27], so the difference in *G. intestinalis* prevalence in different regions may be due to different local climatic conditions, as well as the detection methods, sampling time and sample sizes. Moreover, probably because of the smaller sample sizes, seasonal and age-related correlates previously found in cattle [6] and horses [20] were not found in this study.

Assemblages A and B, responsible for the vast majority of human giardiasis [6, 28], and assemblage E, a common assemblage of *G. intestinalis* in cattle [9], have also been reported in horses [20]. However, perhaps due to the smaller sample sizes, only assemblage B was identified in donkeys in the present study. Assemblage B has a broad host range worldwide [20, 28]. In China, isolates of assemblage B have also been found in non-human primates [2], rabbits [3], horses [17], cattle [29], golden takins (*Budorcas taxicolor bedfordi*) [24], pet chinchillas (*Chinchilla lanigera*) [30], captive wildlife [13], sheep [31] and goats [31], suggesting interspecies transmission of *G. intestinalis* may be commonly occurring in China, and we should pay enough attention to. More importantly, assemblage B was also identified in raw urban wastewater in northern China [32]. Therefore, our results also suggest that donkeys could be a source of giardiasis outbreaks.

Mixed infections of *G. intestinalis* genotypes have been recorded from a wide range of hosts worldwide [29, 33]. In cases of co-infections, some assemblages may be detected preferentially using a single locus primers [34]; thus PCR amplification of a single locus may not reflect the accurate information to *G. intestinalis* infection [34, 35]. A multilocus genotype (MLG) method (*tpi*, *gdh* and *bg* loci) has been developed and widely used for detection of *G. intestinalis* infection [6, 33, 34]. In the present study, 28 *G. intestinalis*-positive samples were also genotyped based on *bg* and *gdh* loci. A total of 28 *tpi*, 22 *bg* and 16 *gdh* gene sequences were obtained, and analysis of these genes revealed only one assemblage (B); however, high genetic polymorphism was observed at these loci within this assemblage (Table 4), implying high genetic diversity of *G. intestinalis* in donkeys in the investigation regions. At the *tpi* locus, seven polymorphic sites were found compared with the GenBank reference sequence AY368169 (Table 4), and four different assemblage B subtypes were identified (Table 4, Additional file 1: Figure S1c). These sequences all represented new subtypes (KU892519–KU892522), and showed a 99% similarity with the reference sequence (accession no. AY368169, from wastewater in USA [36]). Only one subtype was found at the *gdh* locus (Additional file 1: Figure S1b), and the sequence (KU892523) had 99% similarity with the reference sequence available in GenBank with accession number of KR048463 (from a takin in China [24]). Moreover, a total of five SNPs were observed at the *bg* locus (Table 4), and these sequences (KU892516–KU892518) represented three subtypes (Additional file 1: Figure S1a). Moreover, these subtypes were closely clustered with sub-assemblage BIV (Additional file 1: Figure S1), suggesting that sub-assemblage BIV was the most common subtype in donkeys in the investigated regions. Furthermore, phylogenetic analysis of these isolates showed that these isolates exhibit close relationship with isolates from horses, humans and chinchillas, suggesting that transmission of *G. intestinalis* may be occurring among these hosts.

**Table 4:** Variations in *tpi*, *gdh* and *bg* nucleotide sequences among the subtypes of *Giardia intestinalis* assemblage B

Locus	Subtype (*n*)	Nucleotide at position							GenBank ID
*tpi*		10	11	16	182	197	384	525	
	Ref. sequence	C	G	–	G	A	G	G	AY368169
	BIV-1^a^ (*n* = 17)	C	G	–	A	G	A	G	KU892520
	BIV-novel-2 (*n* = 2)	T	C	G	A	G	A	G	KU892519
	BIV-novel-3 (*n* = 6)	T	C	–	A	G	A	G	KU892521
	BIV-novel-4 (*n* = 3)	T	G	–	A	G	A	T	KU892522
*gdh*		219							
	Reference sequence	G							KR048463
	BIV-novel-1 (*n* = 16)	C							KU892523
*bg*		14	179	248	446	447			
	Reference sequence	G	C	C	A	G			KM926514
	BIV-1^b^ (*n* = 12)	G	C	C	G	A			KU892517
	BIV-2^b^ (*n* = 4)	G	T	C	G	A			KU892518
	Bb-7^c^ (*n* = 6)	A	C	T	G	A			KU892516

^a^Identified by Qi et al. [17]

^b^Identified by Coronato Nunes et al. [37]

^c^Identified by Karim et al. [2]

**Fig. 1:**
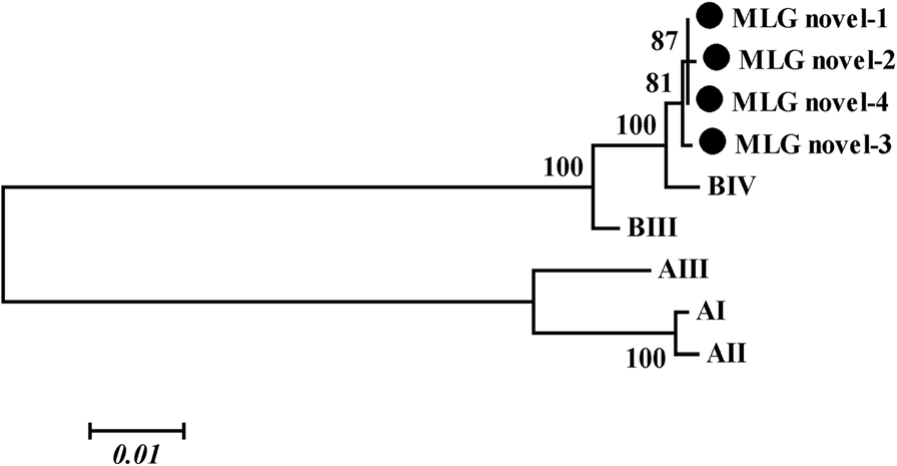
Phylogenetic relationships of *Giardia intestinalis* inferred by the neighbour-joining analysis of concatenated *β-giardin*, *gdh* and *tpi* gene sequences. Multi-locus genotypes from a previous study (Lebbad et al. [38]) are indicated. The bootstrapping was performed using 1000 replicates. Isolates identified in this study are indicated by *solid circles*

Furthermore, 10 out of 28 positive isolates were successfully amplified at all three loci. These samples provided four novel MLGs in the assemblage B, namely MLGs novel-1 to novel-4 (Table 5, Fig. 1). Of these, MLG novel-1 (*n* = 5) was the most prevalent MLG, and responsible for 50% of all MLGs in the present study. These findings suggest a high genetic diversity of this prevalent genotype in donkeys in China, in agreement with previous conclusions that *G. intestinalis* isolates of the same assemblage may be grouped into distinct MLGs [6, 24].

**Table 5:** Multilocus characterization of *Giardia intestinalis* assemblage B isolates from donkeys at *tpi*, *gdh* and *bg* loci

Isolate (*n*)	Genotype	GenBank ID	MLGs
L7 (5)	BIV-1, BIV-novel-1, BIV-1	KU892520, KU892523, KU892516	novel-1
L13 (1)	BIV-novel-2, BIV-novel-1, BIV-2	KU892519, KU892523, KU892517	novel-2
L64 (2)	BIV-novel-3, BIV-novel-1, Bb-7	KU892521, KU892523, KU892518	novel-3
L93 (2)	BIV-1, BIV-novel-1, Bb-7	KU892520, KU892523, KU892518	novel-4

## Conclusions

The present study demonstrated the occurrence of *G. intestinalis* infection in donkeys in China. Sequences analysis suggested that all the *G. intestinalis* isolates represented assemblage B, with four, one and three subtypes of assemblage B at the *tpi gdh* and *bg* loci, respectively. Moreover, four novel MLGs (MLGs novel-1 to novel-4) were identified within assemblage B. The results of the present study not only improve the information of the distribution of *G. intestinalis* genotypes in China, but also provide the foundation data for preventing and controlling *G. intestinalis* infection in donkeys, other animals and humans.

## Additional file

Additional file 1: Figure S1. Phylogenetic tree of *Giardia intestinalis* based on nucleotide sequences of the *β-giardin* (**a**), *gdh* gene (**b**) and *tpi* gene (**c**). Trees were constructed using using the neighbor-joining (NJ) method (Kimura 2-parameter model). Bootstrapping was performed using 1000 replicates. *G. intestinalis* isolates identified in the present study are indicated by solid circles. (TIF 4193 kb)

## Abbreviations

*bg*: Beta giardin gene
CI: Confidence interval
*gdh*: Glutamate dehydrogenase gene
MLG: Multilocus genotype
OR: Odds ratio
*tpi*: Triosephosphate isomerase (*tpi*) gene
