# Supplementary figures and images for: The presence of Giardia intestinalis in donkeys, Equus asinus, in China
Source: Parasit Vectors. 2017 Jan 3;10:3. doi: 10.1186/s13071-016-1936-0 (PMC5209919; doi:10.1186/s13071-016-1936-0)

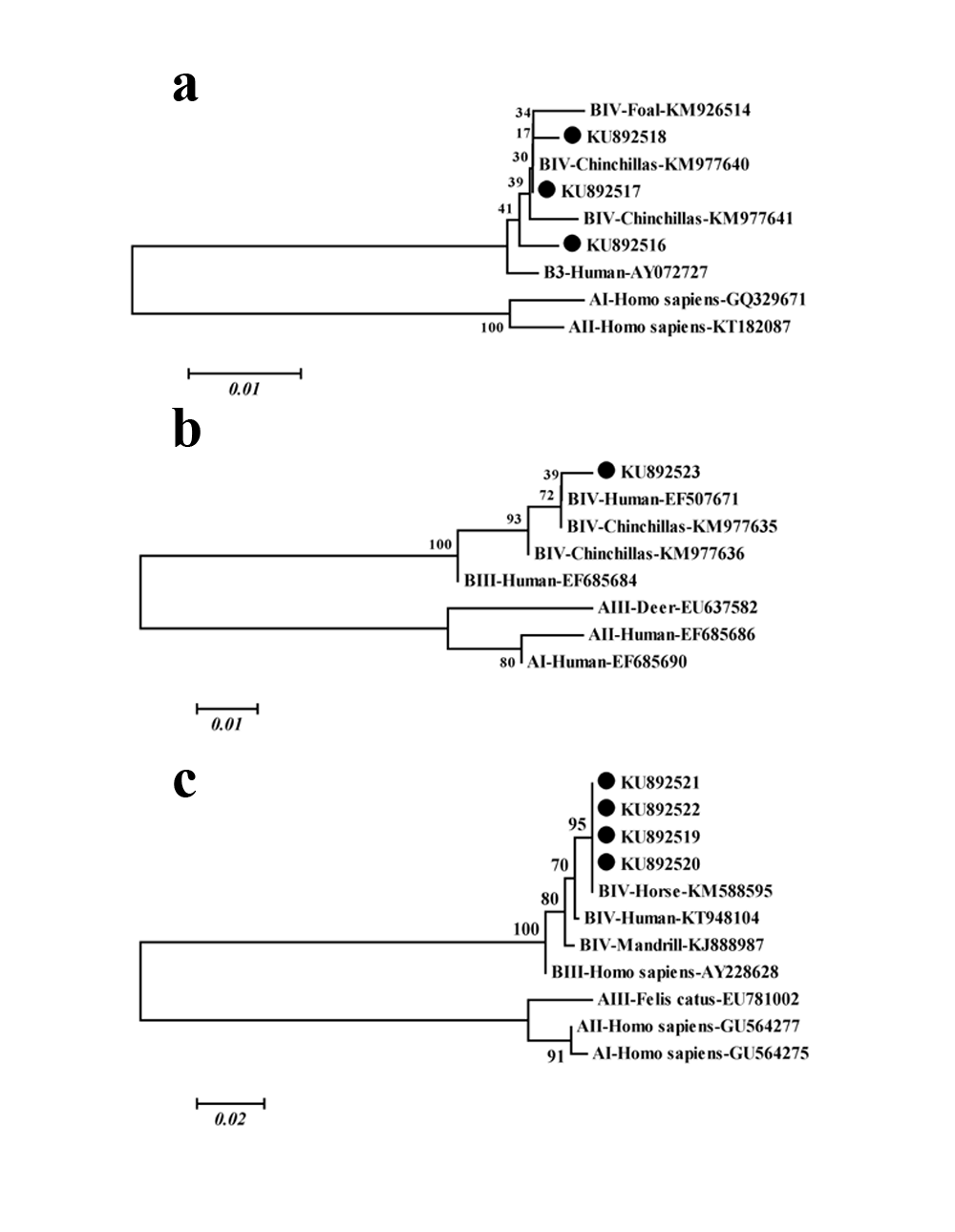

Supplement: Additional file 1: Figure S1. — Phylogenetic tree of Giardia intestinalis based on nucleotide sequences of the β-giardin (a), gdh gene (b) and tpi gene (c). Trees were constructed using using the neighbor-joining (NJ) method (Kimura 2-parameter model). Bootstrapping was performed using 1000 replicates. G. intestinalis isolates identified in the present study are indicated by solid circles. (TIF 4193 kb) [file 13071_2016_1936_MOESM1_ESM.tif]
